# Supplementary material for: Genetic dissection of assortative mating behavior
Source: PLoS Biol. 2019 Feb 7;17(2):e2005902. doi: 10.1371/journal.pbio.2005902 (PMC6366751; doi:10.1371/journal.pbio.2005902)

Genetic dissection of assortative mating behavior

Richard M. Merrill [1,2,3,*], Pasi Rastas[2], Simon H. Martin[2], Maria C. Melo[3,4], Sarah Barker[2], John Davey[2,5], W. Owen McMillan[3] & Chris D. Jiggins[2]

1 Division of Evolutionary Biology, Ludwig-Maximilians-Universität, München, Germany **2** Department of Zoology, University of Cambridge, Cambridge, UK **3** Smithsonian Tropical Research Institute, Panama City, Panama **4** IST Austria, Klosterburg, Austria **5** Department of Biology, University of York, York, UK

*merrill@bio.lmu.de

Supporting Information: Figures S1

Figure S1. QTL analysis of variation of mate preference for individuals with alternative genotypes at LG18@0cM. QTL associated with the proportion of time males court *H. melpomene* (as opposed to *H. cydno*) females on chromosomes 17 for individuals homozygous (i.e. white, CYD:CYD = blue line) and heterozygous (i.e. red, CYD:MEL = orange line) at LG18@0cM. Dashed line represents log odds ratio (LOD) significance threshold (*i.e.* genome-wide alpha = 0.05) for heterozygous (*i.e.* red, CYD:MEL) individuals. Dashes along the x-axis indicate position of genetic markers (SNPs).

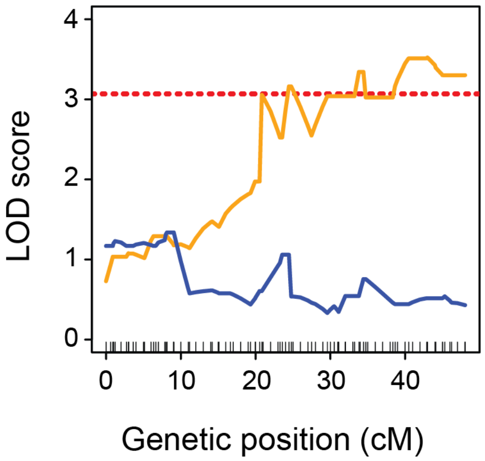

Supplement: S1 Fig — QTL associated with the proportion of time males court H. melpomene (as opposed to H. cydno) females on Chromosome 17 for individuals homozygous (i.e., white, CYD:CYD = blue line) and heterozygous (i.e., red, CYD:MEL = orange line) at LG18@0cM. Dashed line represents LOD significance threshold (i.e., genome-wide alpha = 0.05) for heterozygous (i.e., red, CYD:MEL) individuals. Dashes along the x-axis indicate position of genetic markers (SNPs). CYD, cydno allele; LOD, log odds ratio; MEL, melpomene allele; QTL, quantitative trait locus. (PDF) [file pbio.2005902.s001.pdf]
