# Supplementary material for: Genetic dissection of assortative mating behavior
Source: PLoS Biol. 2019 Feb 7;17(2):e2005902. doi: 10.1371/journal.pbio.2005902 (PMC6366751; doi:10.1371/journal.pbio.2005902)

Genetic dissection of assortative mating behavior

Richard M. Merrill [1,2,3,*], Pasi Rastas[2], Simon H. Martin[2], Maria C. Melo[3,4], Sarah Barker[2], John Davey[2,5], W. Owen McMillan[3] & Chris D. Jiggins[2]

1 Division of Evolutionary Biology, Ludwig-Maximilians-Universität, München, Germany **2** Department of Zoology, University of Cambridge, Cambridge, UK **3** Smithsonian Tropical Research Institute, Panama City, Panama **4** IST Austria, Klosterburg, Austria **5** Department of Biology, University of York, York, UK

*merrill@bio.lmu.de

Supporting Information: Figures S2

Figure S2. Simulations suggest QTL effect sizes are not greatly overestimated. For each simulated effect size, the distribution of all simulated effects (blue) and those which would be significant in our analysis (*i.e.* LOD ≥ 2.99) (orange) are shown. In each case, 'recorded' refers to the empirically measure effect size.

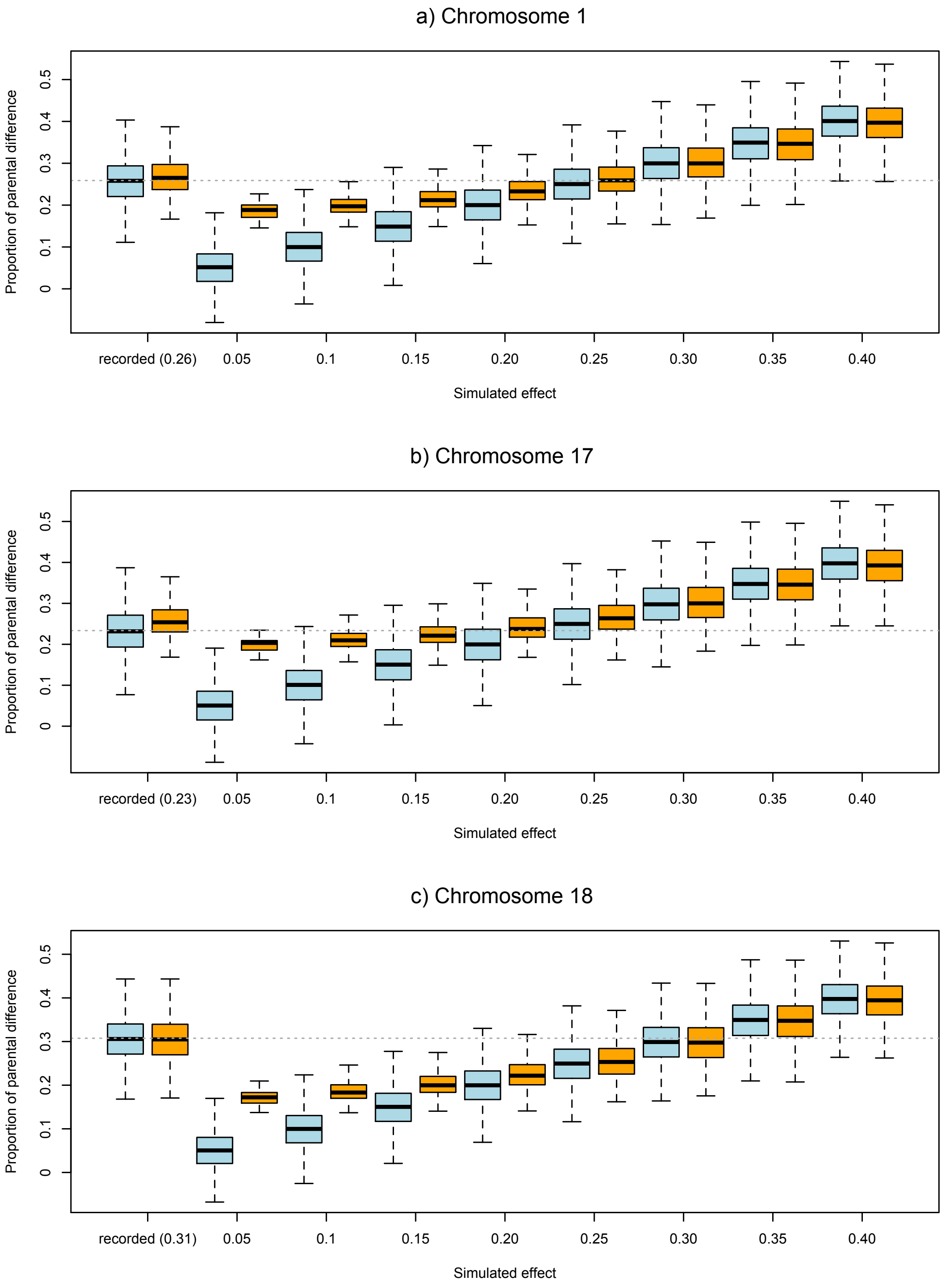

Supplement: S2 Fig — For each simulated effect size, the distribution of all simulated effects (blue) and those which would be significant in our analysis (i.e., LOD ≥ 2.99) (orange) are shown. In each case, “recorded” refers to the empirically measured effect size. LOD, log odds ratio; QTL, quantitative trait locus. (PDF) [file pbio.2005902.s002.pdf]
