# Supplementary material for: Genetic dissection of assortative mating behavior
Source: PLoS Biol. 2019 Feb 7;17(2):e2005902. doi: 10.1371/journal.pbio.2005902 (PMC6366751; doi:10.1371/journal.pbio.2005902)

Genetic dissection of assortative mating behavior

Richard M. Merrill [1,2,3,*], Pasi Rastas[2], Simon H. Martin[2], Maria C. Melo[3,4], Sarah Barker[2], John Davey[2,5], W. Owen McMillan[3] & Chris D. Jiggins[2]

1 Division of Evolutionary Biology, Ludwig-Maximilians-Universität, München, Germany **2** Department of Zoology, University of Cambridge, Cambridge, UK **3** Smithsonian Tropical Research Institute, Panama City, Panama **4** IST Austria, Klosterburg, Austria **5** Department of Biology, University of York, York, UK

*merrill@bio.lmu.de

Supporting Information: Figures S3

Figure S3. Localized levels of admixture (f_d) across all 21 chromosomes. Blue points represent f_d values for 100kb windows. f_d was measured between *H. melpomene rosina* and *H. cydno chioneus* individuals.

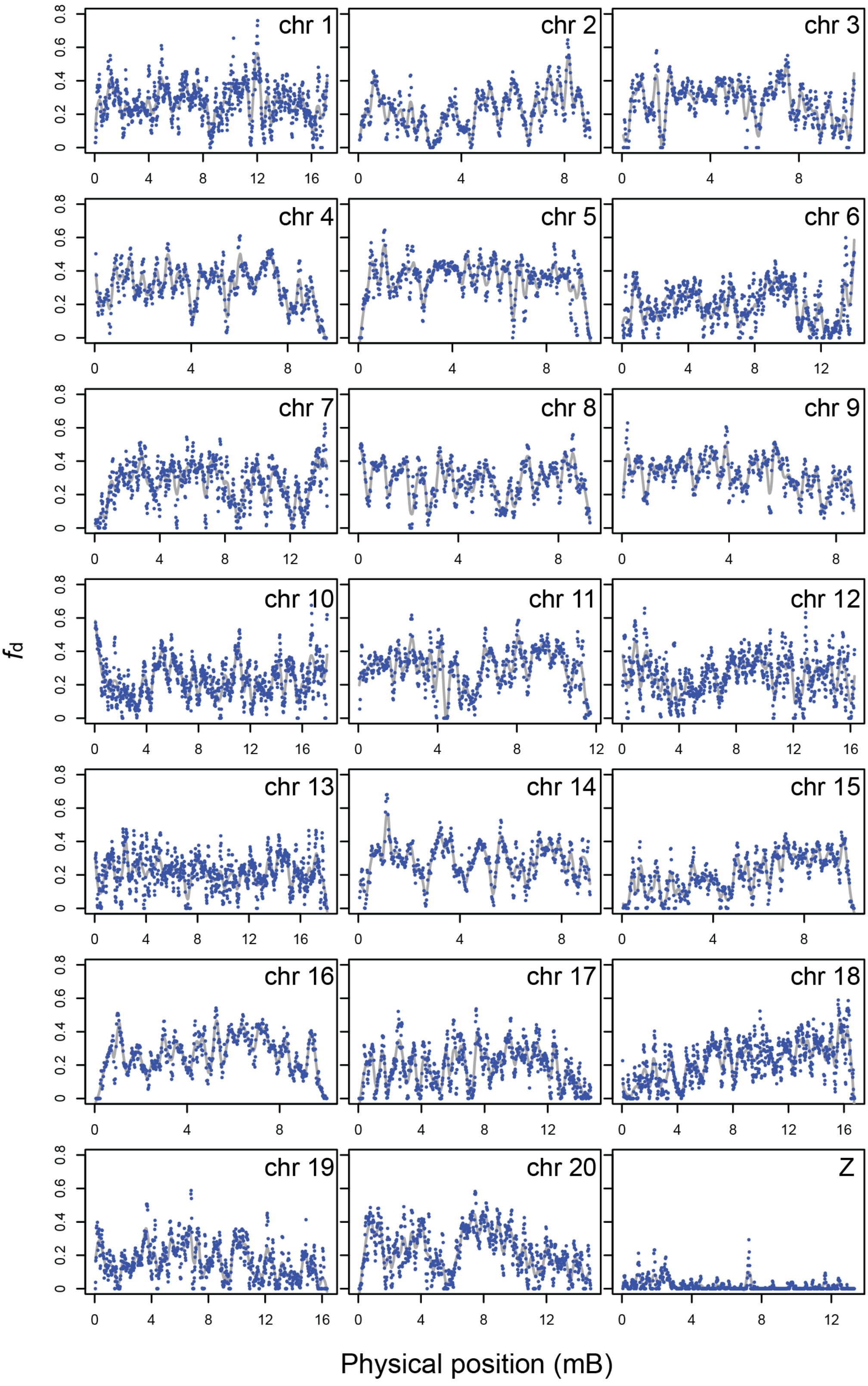

Supplement: S3 Fig — Blue points represent fd values for 100-kb windows. fd was measured between H. melpomene rosina and H. cydno chioneus individuals. (PDF) [file pbio.2005902.s003.pdf]
