# Supplementary material for: Genetic dissection of assortative mating behavior
Source: PLoS Biol. 2019 Feb 7;17(2):e2005902. doi: 10.1371/journal.pbio.2005902 (PMC6366751; doi:10.1371/journal.pbio.2005902)

Genetic dissection of assortative mating behavior

Richard M. Merrill [1,2,3,*], Pasi Rastas[2], Simon H. Martin[2], Maria C. Melo[3,4], Sarah Barker[2], John Davey[2,5], W. Owen McMillan[3] & Chris D. Jiggins[2]

1 Division of Evolutionary Biology, Ludwig-Maximilians-Universität, München, Germany **2** Department of Zoology, University of Cambridge, Cambridge, UK **3** Smithsonian Tropical Research Institute, Panama City, Panama **4** IST Austria, Klosterburg, Austria **5** Department of Biology, University of York, York, UK

*merrill@bio.lmu.de

Supporting Information: Figures S5

Figure S5. Decline in linkage disequilibrium (LD) of linked and unlinked loci under an assumption of random mating. Whereas linkage disequilibrium (*D*) between unlinked loci (red solid line) declines by 50% in one generation of random mating, LD between two loci that are 1.2cM apart would decline by only 1.2 % per generation (black solid line), and LD between two loci that are 4.8cM apart (gray dashed line) would decline by only 4.6% per generation.

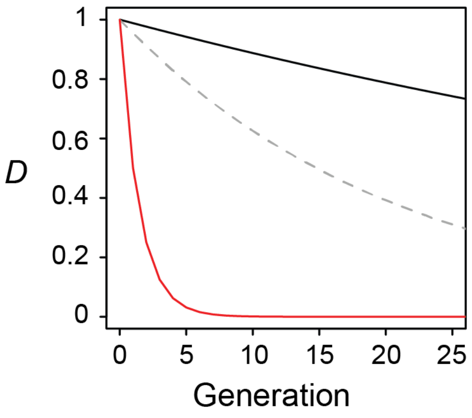

Supplement: S5 Fig — Whereas LD (D) between unlinked loci (red solid line) declines by 50% in one generation of random mating, LD between two loci that are 1.2 cM apart would decline by only 1.2% per generation (black solid line), and LD between two loci that are 4.8 cM apart (gray dashed line) would decline by only 4.6% per generation. cM, centiMorgan; LD, linkage disequilibrium. (PDF) [file pbio.2005902.s005.pdf]
