# Supplementary material for: Genetic dissection of assortative mating behavior
Source: PLoS Biol. 2019 Feb 7;17(2):e2005902. doi: 10.1371/journal.pbio.2005902 (PMC6366751; doi:10.1371/journal.pbio.2005902)
Supplement: S2 Table — GLMM, generalized linear mixed model; QTL, quantitative trait locus. (PDF) [file pbio.2005902.s007.pdf]

Genetic dissection of assortative mating behavior

Richard M. Merrill [1,2,3,*], Pasi Rastas[2], Simon H. Martin[2], Maria C. Melo[3,4], Sarah Barker[2], John Davey[2,5], W. Owen McMillan[3] & Chris D. Jiggins[2]

1 Division of Evolutionary Biology, Ludwig-Maximilians-Universität, München, Germany **2** Department of Zoology, University of Cambridge, Cambridge, UK **3** Smithsonian Tropical Research Institute, Panama City, Panama **4** IST Austria, Klosterburg, Austria **5** Department of Biology, University of York, York, UK

*merrill@bio.lmu.de

Supporting information: Table S7

Table S7. Summary of genome-wide QTL analyses using binomial GLMM methods (reported in main text) and non-parametric methods implemented in R/qtl.

	**Binomial GLMM**			**Non-parametric**		
Chromosome	Position (cM)	LOD	*P*	Position (cM)	LOD	*P*
1	4.23	4.54	<0.001	4.23	3.6	~0.009
17	24.47	3.5	~0.013	24.47	2.89	~0.049
18	0	6.83	<0.001	0	5.34	<0.001

Position in cM refers to the position the peak LOD score, i.e. the most likely genetic position of the putative QTL, for each QTL. *P* values are determined through permutation as described in the methods.
